# Immune checkpoint inhibitors and myocarditis–myositis–myasthenia gravis overlap: a FAERS pharmacovigilance study with time-to-onset characterization

**DOI:** 10.3389/fphar.2026.1825269

**Published:** 2026-05-18

**Authors:** Burak Pacaci, Bilal Yildirim, Mustafa Alperen Tunc, Ahmet Demirel, Firat Akagunduz, Erkam Kocaaslan, Ali Kaan Guren, Nazim Can Demircan, Ibrahim Vedat Bayoglu

**Affiliations:** 1 Department of Medical Oncology, Marmara University Pendik Training and Research Hospital, Istanbul, Türkiye; 2 Department of Internal Medicine, Marmara University Pendik Training and Research Hospital, Istanbul, Türkiye

**Keywords:** FAERS, immune checkpoint inhibitors, myasthenia gravis, myocarditis, myositis, pharmacovigilance, time-to-onset

## Abstract

**Background:**

Immune checkpoint inhibitors (ICIs) are associated with rare but life-threatening immune-related adverse events. Myocarditis–myositis–myasthenia gravis (MMM) overlap is among the most severe neuromuscular–cardiac toxicity patterns, but population-scale data on disproportionality, reported death outcomes, and time-to-onset (TTO) remain limited.

**Methods:**

We conducted a retrospective pharmacovigilance study using the FDA Adverse Event Reporting System (FAERS) from 2015Q1 to 2025Q4. Strict MMM overlap was defined at the report level by co-reporting of the Preferred Terms “myocarditis,” “myositis,” and “myasthenia gravis”. ICI exposure was restricted to selected PD-1, PD-L1, and CTLA-4 inhibitors recorded as primary or secondary suspect drugs (PS/SS), with a prespecified sensitivity analysis restricted to PS reports only. Disproportionality was assessed primarily using reporting odds ratios (RORs) with Haldane–Anscombe correction for sparse cells; the Information Component (IC) and IC025 were calculated as complementary Bayesian metrics. Drug-level TTO was estimated by linking therapy start dates and event dates within eligible reports.

**Results:**

Among 15,187,311 deduplicated FAERS reports, 151 met the strict MMM overlap definition, including 131 ICI-exposed reports. ICI exposure showed a strong disproportionality signal for strict MMM overlap (ROR 397.29, 95% CI 249.44–632.76; IC 5.50, IC025 5.24). Reported death outcomes were observed in 49/131 (37.4%) ICI-exposed MMM overlap reports, including 31/80 (38.8%) in PD-1 reports, 5/16 (31.3%) in PD-L1 reports, and 13/35 (37.1%) in combination ICI reports. Drug-level TTO was available for 53 ICI-exposed reports, with a median of 25 days (IQR 18–38); restricting to TTO ≤365 days yielded a similar median of 24 days (IQR 18–37), supporting a predominantly early-onset pattern. Sensitivity analyses restricted to PS were directionally consistent.

**Conclusion:**

In FAERS, ICI-associated strict MMM overlap showed a strong disproportionality signal and predominantly early reported onset; reported death outcomes were also observed in a substantial proportion of ICI-exposed overlap reports. These findings support heightened clinical vigilance for cardio-neuromuscular overlap toxicity, particularly during the first months after ICI initiation.

## Highlights

- Reports to the FDA’s safety database suggest that a rare but serious combination of heart inflammation, muscle inflammation, and severe muscle weakness is reported far more often in people receiving immune checkpoint inhibitors than in those receiving other medicines.
- When this overlap is reported, it tends to happen early after starting treatment, and a substantial proportion of reports also included reported death outcomes in FAERS, highlighting the need for rapid recognition and urgent care.
- Similar patterns across different checkpoint inhibitor types suggest this is a treatment-class safety concern, so patients and clinicians should be alert to new chest symptoms, shortness of breath, severe fatigue, muscle pain/weakness, or drooping eyelids—especially in the first weeks.

## 1 Introduction

Immune checkpoint inhibitors (ICIs) have transformed the treatment landscape of multiple malignancies, but their growing use has been accompanied by a broad spectrum of immune-related adverse events (irAEs) affecting nearly every organ system. Among these toxicities, cardiovascular and neuromuscular irAEs are uncommon but clinically important because of their potential for rapid deterioration and severe outcomes (Postow et al., 2018; Wolchok et al., 2013).

ICI-related myocarditis is rare but has been repeatedly associated with substantial severity, particularly when accompanied by inflammatory skeletal muscle involvement or neuromuscular junction dysfunction (Mahmood et al., 2018; Moslehi et al., 2018). Within this spectrum, myocarditis–myositis–myasthenia gravis (MMM) overlap has emerged as a particularly severe cardio-neuromuscular phenotype. Recent evidence from systematic reviews, case series, and retrospective cohorts suggests that this overlap syndrome often develops early after ICI initiation and may be associated with a high burden of serious clinical outcomes; however, the literature remains dominated by pooled case reports, small institutional experiences, or studies focused primarily on myocarditis or neuromuscular manifestations rather than the strict triad phenotype itself (Suzuki et al., 2017; Pathak et al., 2021; Johnson et al., 2016; Lipe et al., 2024; Sánchez-Camacho et al., 2025; Itzhaki Ben Zadok et al., 2025; Plomp et al., 2025).

Large spontaneous reporting systems, such as the FDA Adverse Event Reporting System (FAERS), provide an opportunity to evaluate rare and severe toxicities at scale. Although FAERS cannot establish incidence or causality, disproportionality analyses can identify whether a specific adverse-event pattern is reported disproportionately often with a given drug exposure within the reporting environment (van Puijenbroek et al., 2002; Montastruc et al., 2011). In parallel, time-to-onset (TTO) analyses may help characterize the temporal clustering of reported events and inform clinical surveillance windows.

In this study, we used FAERS data from 2015Q1 to 2025Q4 to characterize ICI-associated strict MMM overlap, defined by the co-reporting of myocarditis, myositis, and myasthenia gravis within the same report. Our objectives were to quantify disproportionality overall and by ICI class, describe reported death outcomes among overlap reports, and characterize drug-level TTO patterns in reports with usable timing data. By focusing on a high-specificity triad phenotype, this study aimed to provide population-scale pharmacovigilance context for a rare but clinically consequential ICI toxicity pattern.

## 2 Methods

### 2.1 Data source and study design

We conducted a retrospective pharmacovigilance study using quarterly FAERS data from 2015Q1 through 2025Q4. Core tables used for this analysis were DEMO, DRUG, REAC, THER, and OUTC. To minimize duplicate counting, reports were deduplicated at the case level by retaining the most recent version (highest CASEVERSION) for each CASEID before analysis. Because FAERS is a publicly available de-identified database, institutional review board approval and informed consent were not required.

### 2.2 Case definition: strict MMM overlap

The primary outcome was strict MMM overlap, defined at the report level by co-reporting of all three MedDRA Preferred Terms (PTs) within the same FAERS report: “Myocarditis,” “Myositis,” and “Myasthenia gravis.” We used PTs as recorded in the raw quarterly FAERS files without remapping across MedDRA versions. This definition was selected to prioritize phenotype specificity for the triad syndrome, recognizing that it may underascertain clinically related but less explicitly coded cases.

### 2.3 Exposure definition and ICI classification

The exposure of interest was a prespecified set of PD-1, PD-L1, and CTLA-4 ICIs identified using brand-safe matching of generic and proprietary names. To improve attribution within the spontaneous reporting setting, the primary analysis restricted exposure to drugs coded as primary suspect or secondary suspect (PS/SS) in the DRUG table. A prespecified sensitivity analysis further restricted exposure to PS reports only.

Included agents were nivolumab, pembrolizumab, and cemiplimab (PD-1); atezolizumab, durvalumab, and avelumab (PD-L1); and ipilimumab and tremelimumab (CTLA-4). Reports containing no included ICI under the applied exposure definition were classified as Non-ICI. Reports containing two or more different ICI classes within the same report were classified as combination ICI (Combo). All other ICI-exposed reports were assigned to a single class according to the class represented within that report. Because FAERS does not reliably capture treatment sequencing, this report-level classification reflects co-reporting rather than confirmed concurrent administration.

### 2.4 Disproportionality analysis

Disproportionality was assessed primarily using the reporting odds ratio (ROR), comparing the odds of strict MMM overlap in ICI-exposed versus Non-ICI reports within the same FAERS reporting universe. In spontaneous reporting systems, disproportionality metrics such as the ROR are used to assess whether a drug–event pair is reported disproportionately often relative to the reporting background, rather than to estimate incidence or causal risk (van Puijenbroek et al., 2002; Montastruc et al., 2011). RORs and 95% confidence intervals were derived from standard 2 × 2 contingency tables. Because strict MMM overlap is rare and some class-specific cells were sparse, we prespecified the Haldane–Anscombe continuity correction (0.5 added to all cells) to avoid unstable or infinite estimates.

A positive frequentist signal was defined as a lower 95% confidence limit of the ROR greater than 1 with at least three exposed strict MMM overlap reports. As a complementary Bayesian robustness measure, we also calculated the Information Component (IC) and its lower 95% credibility limit (IC025), with IC025 > 0 considered supportive of a positive Bayesian signal. ROR was treated as the primary disproportionality metric, and IC as a secondary confirmatory measure.

Class-specific analyses compared each ICI class separately against the same Non-ICI reference group. Therefore, class-specific denominators differ across comparisons and should not be summed across ICI classes.

### 2.5 Reported death outcomes

Severity was described using the FAERS OUTC code “DE” (death), hereafter termed reported death outcome. Reported death outcome proportions were summarized within strict MMM overlap reports by exposure group. Because FAERS does not provide validated cause-of-death adjudication, these data were interpreted as report-level death outcomes rather than disease-specific or treatment-attributable mortality.

### 2.6 Event date derivation and drug-level TTO

For TTO analyses, we derived a single event date using a prespecified hierarchy of available FAERS date fields, prioritizing EVENT_DT, followed by MFR_DT, INIT_FDA_DT, and FDA_DT. When only partial dates were available, missing components were imputed to the midpoint of the month or year to enable interval calculation.

Drug-level TTO was estimated among ICI-exposed strict MMM overlap reports by linking DRUG and THER records within each report using the report identifier and drug-sequence fields. For each eligible report, the index ICI start date was defined as the earliest valid therapy start date among included ICIs meeting the applied exposure definition. TTO was calculated as the interval in days from this start date to the derived event date. Descriptive TTO summaries were restricted to non-negative intervals. To reduce distortion from rare extreme late-reported onsets, we also prespecified a restricted descriptive analysis for TTO ≤365 days.

### 2.7 Temporal pattern analysis

To describe the overall shape of the reported onset-time distribution, we performed a descriptive Weibull analysis using case-only TTO data. Because FAERS lacks treatment denominators and longitudinal follow-up, this analysis was interpreted as a characterization of the distribution of reported onset times rather than an estimate of population-level hazard. We report these findings as supportive temporal descriptors rather than as primary evidence.

### 2.8 Sensitivity analyses

All primary analyses were repeated using the more restrictive PS-only exposure definition to evaluate robustness to stricter suspect attribution.

### 2.9 Statistical analysis

All analyses were performed in R (R Foundation for Statistical Computing, Vienna, Austria). Disproportionality results are reported as RORs with 95% confidence intervals, alongside complementary IC metrics. TTO is summarized using medians and interquartile ranges. Statistical tests were two-sided, and p values were interpreted cautiously given the exploratory nature of the analysis.

## 3 Results

### 3.1 Reporting universe and case identification

**FIGURE 1:**
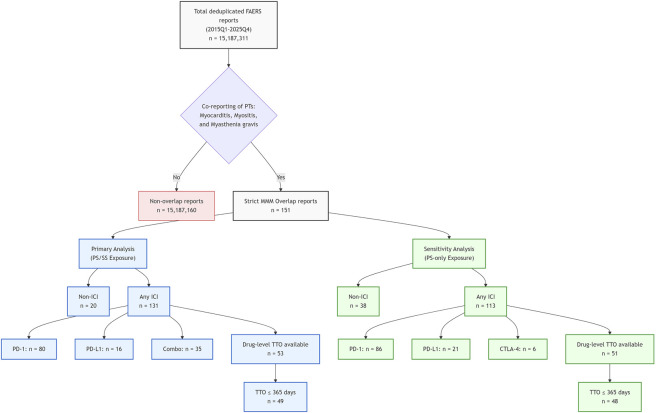
Study flow diagram of case selection and classification. Notes: Deduplicated FAERS reports (2015Q1–2025Q4) were filtered for strict MMM overlap and categorized by ICI exposure. The primary analysis defined exposure using primary/secondary suspect roles (PS/SS), while the sensitivity analysis was restricted to primary suspect only (PS-only). Abbreviations: FAERS, FDA Adverse Event Reporting System; MMM, myocarditis–myositis–myasthenia gravis; ICI, immune checkpoint inhibitor; PS, primary suspect; SS, secondary suspect; TTO, time-to-onset.

Between 2015Q1 and 2025Q4, a total of 15,187,311 deduplicated reports were identified in FAERS. Applying the predefined strict case definition yielded 151 reports meeting criteria for concurrent myocarditis, myositis, and myasthenia gravis. Of these, 131 reports (86.8%) were classified as ICI-exposed under the primary PS/SS definition, whereas 20 reports occurred in the Non-ICI population (Figure 1).

Demographic characteristics, reporter type, reporting region, and serious outcomes of ICI-exposed MMM overlap reports are summarized in Supplementary Table S5. Among ICI-exposed MMM overlap reports, hospitalization and life-threatening outcomes were reported in 74.0% and 40.5% of cases, respectively. Annual report counts increased over the study period (Supplementary Figure S1), a pattern that may reflect expanding ICI use, improved clinical recognition, and reporting-related factors rather than a direct estimate of incidence.

### 3.2 Disproportionality of strict MMM overlap

ICI exposure was associated with a markedly disproportionate reporting of strict MMM overlap relative to the Non-ICI FAERS reporting universe. In the primary PS/SS analysis, the ROR was 397.29 (95% CI 249.44–632.76). The complementary Bayesian analysis was directionally consistent (IC 5.50; IC025 5.24) (Table 1).

**TABLE 1:** Disproportionality of strict MMM overlap reports for ICIs in FAERS (2015Q1–2025Q4; PS/SS exposure).

Exposure	MMM overlap	Non-overlap	Total	ROR (95% CI)	IC (IC025)
Any ICI (PS/SS)	a = 131	b = 241,315	241,446	397.29 (249.44–632.76)	5.50 (5.24)
Non-ICI	c = 20	d = 14,945,845	14,945,865	Reference	Reference
Total	151	15,187,160	15,187,311	​	​

CI, confidence interval; FAERS, FDA adverse event reporting system; ICI, immune checkpoint inhibitor; MMM, myocarditis–myositis–myasthenia gravis overlap; PS, primary suspect; PT, preferred term; ROR, reporting odds ratio; SS, secondary suspect; IC, information component; IC025, lower 95% credibility limit of IC.

Class-specific analyses also showed strong signals across ICI categories, with the largest signal observed for Combo reports (ROR 783.34, 95% CI 454.75–1,349.34), followed by PD-1 inhibitors (ROR 394.65, 95% CI 243.01–640.93) and PD-L1 inhibitors (ROR 230.92, 95% CI 120.76–441.58). No CTLA-4 monotherapy MMM overlap reports were observed under the PS/SS definition. Sensitivity analyses restricted to primary suspect reports yielded attenuated but still robust signals, supporting the stability of the primary finding under stricter exposure attribution (Supplementary Tables S2, S4).

### 3.3 Reported death outcomes

**FIGURE 2:**
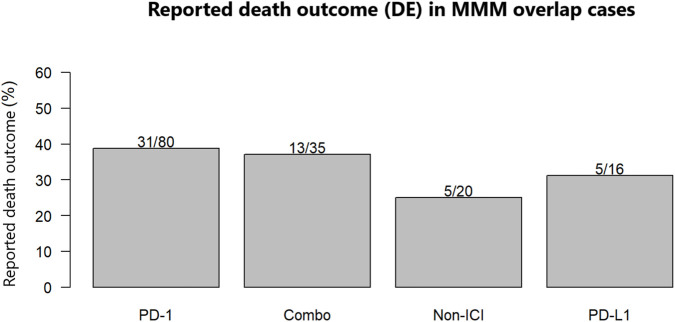
Reported death outcome (DE) proportions among MMM overlap reports by exposure group. Abbreviations: DE, death.

Reported death outcomes were observed in a substantial proportion of strict MMM overlap reports. In the PS/SS analysis, reported death outcomes occurred in 49 of 131 ICI-exposed reports (37.4%), including 31 of 80 PD-1 reports (38.8%), five of 16 PD-L1 reports (31.3%), and 13 of 35 Combo reports (37.1%). The corresponding proportion in the Non-ICI group was five of 20 reports (25.0%) (Figure 2). Similar patterns were observed in the PS-only sensitivity analysis (Supplementary Table S3). Because FAERS does not provide validated cause-of-death adjudication, these findings should be interpreted as report-level death outcomes rather than treatment-attributable or disease-specific mortality.

### 3.4 Drug-level TTO

**FIGURE 3:**
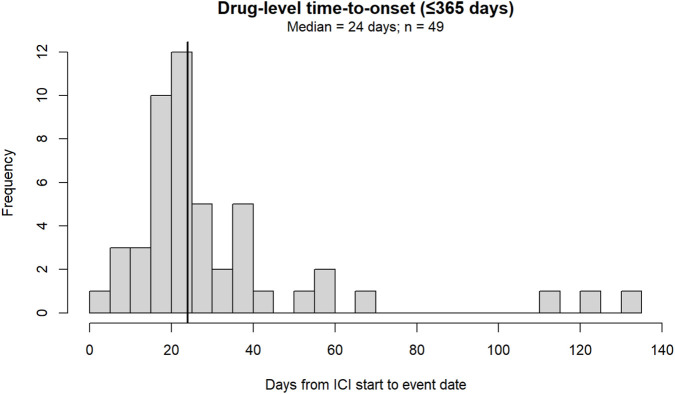
Distribution of drug-level TTO among ICI-exposed MMM overlap reports restricted to ≤365 days.

Drug-level TTO was available for 53 ICI-exposed strict MMM overlap reports under the PS/SS definition. The median TTO was 25 days (IQR 18–38), with a range of 4 to 1,674 days. Among 53 evaluable ICI-exposed reports, 34 (64.1%) occurred within 30 days, 45 (84.9%) within 60 days, and 46 (86.8%) within 90 days after ICI initiation. Because the distribution was strongly right-skewed by rare late-onset reports, we also performed a prespecified restricted descriptive analysis for TTO ≤365 days. In that window, 49 reports remained, with a median TTO of 24 days (IQR 18–37) and a maximum of 131 days (Figure 3). Taken together, these data indicate that most evaluable reported onsets clustered within the first months after ICI initiation, while a small number of extreme late reports extended the right tail of the distribution.

### 3.5 Descriptive temporal pattern

In descriptive Weibull analyses, the estimated shape parameter was 0.65 (95% CI 0.56–1.04) for all eligible TTO values and 1.39 (95% CI 1.20–2.01) after restriction to TTO ≤365 days (Table 2). Given the absence of exposure denominators and longitudinal follow-up in FAERS, these estimates should be interpreted only as descriptors of the distribution of reported onset times rather than as population-level hazard measures. The difference between unrestricted and restricted analyses likely reflects the influence of rare extreme late-onset reports on the overall distribution. PS-only sensitivity analyses yielded similar results (Supplementary Table S2).

**TABLE 2:** Drug-level TTO summaries and Weibull shape (β) estimates among ICI-exposed strict MMM overlap reports.

Dataset	n	Median (days)	IQR (days)	Min–Max (days)	Weibull β (bootstrap 95% CI)
All TTO	53	25	18–38	4–1674	0.65 (0.56–1.04)
TTO ≤365 days	49	24	18–37	4–131	1.39 (1.20–2.01)

CI, confidence interval; IQR, interquartile range; TTO, time-to-onset.

## 4 Discussion

In this large FAERS pharmacovigilance study, ICI exposure was associated with a very strong disproportionality signal for strict MMM overlap. Reported death outcomes were observed in more than one-third of ICI-exposed overlap reports, and, among reports with usable timing data, drug-level onset clustered early after treatment initiation. Taken together, these findings support strict MMM overlap as a rare but high-severity cardio-neuromuscular immune-related toxicity pattern within the FAERS reporting environment (Mahmood et al., 2018; Moslehi et al., 2018; Suzuki et al., 2017; Pathak et al., 2021; Lipe et al., 2024; Salem et al., 2018; Lyon et al., 2018; Ganatra and Neilan, 2018).

The strength and internal consistency of the signal across PD-1, PD-L1, and Combo categories argue against an isolated drug-specific anomaly and are more compatible with a broader class-associated immune toxicity pattern. The highest disproportionality signal was observed in Combo reports, which is directionally consistent with the known tendency of multi-checkpoint blockade to intensify immune activation and increase the frequency and severity of irAEs (Wolchok et al., 2013; Johnson et al., 2016; Larkin et al., 2015). Mechanistically, this overlap phenotype is biologically plausible because checkpoint blockade may promote cross-reactive immune injury involving shared striated-muscle antigens in cardiac muscle, skeletal muscle, and the neuromuscular junction (Postow et al., 2018; Johnson et al., 2016; Lyon et al., 2018; Ganatra and Neilan, 2018).

Our findings fit within, and extend, the current clinical literature. A 2024 systematic review of ICI-associated MMM overlap reported an in-hospital mortality of 38.0% and a median onset of 21 days, which is notably close to the 37.4% reported death outcome proportion and 25-day median TTO observed in our ICI-exposed FAERS cohort (Lipe et al., 2024). At the same time, that review was based predominantly on case reports and small case series, whereas our study provides population-scale pharmacovigilance context for the strict triad phenotype. In this sense, our analysis complements rather than replaces the existing clinical literature by showing that a highly specific MMM overlap pattern is reported disproportionately often with ICI exposure in the broader FAERS reporting universe (Lipe et al., 2024; van Puijenbroek et al., 2002; Montastruc et al., 2011). Selected published clinical studies on ICI-associated MMM overlap and related overlap-spectrum phenotypes are summarized in Supplementary Table S6.

More recent multicentric retrospective cohort data further indicate that ICI-related myositis and myasthenic features frequently overlap in clinical practice, supporting the view that this toxicity spectrum extends beyond isolated anecdotal case reports (Plomp et al., 2025). Notably, this phenotype has also been reported in gynecologic oncology, including a recent dostarlimab-associated endometrial cancer case, suggesting that MMM overlap is not confined to melanoma- or lung cancer-dominant settings (Abozenah et al., 2025).

Prior myocarditis-focused pharmacovigilance and observational studies also provide useful context for interpreting the severity of overlap phenotypes. ICI-related myocarditis alone has been associated with substantial clinical severity and reported fatal outcomes, while overlap with myositis or myasthenia gravis has repeatedly been linked to worse clinical trajectories (Mahmood et al., 2018; Moslehi et al., 2018; Pathak et al., 2021; Salem et al., 2018; Lyon et al., 2018; Ganatra and Neilan, 2018; D'Souza et al., 2021; Zamami et al., 2019; Reeves et al., 2025). In a recent cohort of ICI-related myocarditis, concomitant immune-related myopathy was associated with a greater likelihood of significant arrhythmias, supporting the concept that overlap phenotypes may carry a particularly unstable cardiologic profile (Itzhaki Ben Zadok et al., 2025). From a pathophysiologic perspective, concomitant myocardial inflammation, skeletal muscle involvement, and neuromuscular junction dysfunction may create a compounded risk profile in which arrhythmia or conduction abnormalities coexist with respiratory or bulbar compromise, plausibly contributing to adverse outcomes in this syndrome (Pathak et al., 2021; Lipe et al., 2024; Itzhaki Ben Zadok et al., 2025; Lyon et al., 2018; Ganatra and Neilan, 2018).

The temporal pattern observed in our study also carries a clinically actionable message. Even after restricting the analysis to TTO ≤365 days, the median onset remained approximately 3–4 weeks, indicating that reported MMM overlap most often emerges early after ICI initiation. This is consistent with prior reports suggesting that overlap syndromes tend to occur early in the treatment course, particularly during the first weeks to months after exposure (Mahmood et al., 2018; Moslehi et al., 2018; Suzuki et al., 2017; Pathak et al., 2021; Lipe et al., 2024). At the same time, the presence of rare extreme late-onset reports in our dataset suggests that clinical suspicion should not be limited exclusively to the earliest infusion period. This was further supported by the finding that 64.1%, 84.9%, and 86.8% of evaluable reports occurred within 30, 60, and 90 days after ICI initiation, respectively.

The increase in annual MMM overlap reports over time deserves careful interpretation. This trend likely reflects several overlapping factors, including expanding ICI use across malignancies and treatment settings, improving recognition of overlap syndromes by oncologists, cardiologists, and neurologists, and increased pharmacovigilance awareness with consequent stimulated reporting (Moslehi et al., 2018; van Puijenbroek et al., 2002; Montastruc et al., 2011; Salem et al., 2018; Reeves et al., 2025). Because FAERS lacks exposure denominators and is inherently sensitive to reporting behavior, this temporal increase should not be interpreted as a direct estimate of rising incidence.

From a clinical perspective, MMM overlap should prompt urgent multidisciplinary evaluation. Although standardized treatment algorithms remain incompletely defined, the available literature consistently supports immediate interruption of ICI therapy, prompt cardiac and neuromuscular assessment—including early ECG, troponin, creatine kinase testing, and prompt co-management by cardiology and neurology teams—and early initiation of high-dose corticosteroids when overlap toxicity is suspected (Suzuki et al., 2017; Pathak et al., 2021; Lipe et al., 2024; Sánchez-Camacho et al., 2025; Lyon et al., 2018; Ganatra and Neilan, 2018; Furlepa et al., 2025). In more severe or myasthenia-predominant presentations, intravenous immunoglobulin or plasma exchange is often considered, particularly when respiratory, bulbar, or fulminant cardiac manifestations are present (Suzuki et al., 2017; Pathak et al., 2021; Lipe et al., 2024; Sánchez-Camacho et al., 2025; Furlepa et al., 2025). Contemporary reports also emphasize that management remains heterogeneous, with limited consensus regarding steroid dose, treatment duration, escalation thresholds, and the role of second- or third-line immunomodulatory therapies; accordingly, treatment decisions are still guided primarily by retrospective cohorts, pooled case-based evidence, and expert practice patterns rather than standardized prospective algorithms (Sánchez-Camacho et al., 2025; Furlepa et al., 2025). In this context, our findings help bridge pharmacovigilance data to bedside practice by reinforcing the need for a low threshold for integrated cardio-neuromuscular evaluation in symptomatic patients receiving ICIs.

Several limitations inherent to spontaneous reporting systems should be emphasized. First, disproportionality analysis estimates reporting odds rather than incidence or causal risk and is susceptible to underreporting, stimulated reporting, confounding by indication, and differential reporting intensity (van Puijenbroek et al., 2002; Montastruc et al., 2011). Because the comparator was the overall Non-ICI FAERS reporting universe, the magnitude of disproportionality may also be influenced by oncology-specific reporting intensity, confounding by indication, and background seriousness differences between ICI-exposed and Non-ICI reports. Second, our strict PT-based case definition intentionally prioritized specificity, but this likely reduced sensitivity and may have missed clinically compatible cases coded less explicitly. Because this strict PT-based definition likely enriched for explicitly recognized overlap presentations, the observed reported death outcome burden may not be fully generalizable to the broader spectrum of ICI-related overlap toxicity. Third, FAERS does not permit adjudication of diagnosis severity, biologic plausibility, or cause of death; therefore, our findings regarding death should be interpreted strictly as reported death outcomes rather than treatment-attributable or disease-specific mortality. FAERS also does not permit reliable classification of which component of the overlap phenotype was the dominant driver of deterioration or death in individual cases. Because the present analysis was restricted to the strict triad phenotype, we also could not perform a meaningful within-dataset comparison of lethality across myocarditis-predominant, myositis-predominant, or myasthenia-predominant overlap presentations. Fourth, drug-level TTO estimation was feasible only in a subset of reports with usable date linkage, and rare extreme late reports may reflect delayed recognition, reporting imprecision, or complex treatment sequencing. Finally, because exposure classification was restricted to selected checkpoint inhibitors and suspect roles, some relevant reports may have been misclassified, likely biasing estimates toward the null.

In conclusion, this FAERS study shows that ICI exposure is associated with a strong disproportionality signal for strict MMM overlap, that reported onset clusters predominantly in the early treatment period, and that reported death outcomes were observed in a substantial proportion of ICI-exposed overlap reports. Although these findings should not be interpreted as incidence estimates or treatment-attributable mortality, they reinforce MMM overlap as a rare but clinically serious immune-related toxicity phenotype that warrants early recognition, multidisciplinary assessment, and aggressive initial management when suspected (Mahmood et al., 2018; Moslehi et al., 2018; Suzuki et al., 2017; Pathak et al., 2021; Lipe et al., 2024; Sánchez-Camacho et al., 2025; Lyon et al., 2018; Ganatra and Neilan, 2018; Furlepa et al., 2025).

## Data Availability

The datasets presented in this study can be found in online repositories. The names of the repository/repositories and accession number(s) can be found below: https://www.fda.gov/drugs/fdas-adverse-event-reporting-system-faers/fda-adverse-event-reporting-system-faers-public-dashboard.
