# Supplementary material for: Immune checkpoint inhibitors and myocarditis–myositis–myasthenia gravis overlap: a FAERS pharmacovigilance study with time-to-onset characterization
Source: Front Pharmacol. 2026 May 18;17:1825269. doi: 10.3389/fphar.2026.1825269 (PMC13222775; doi:10.3389/fphar.2026.1825269)
Supplement: Supplementary file 1 [file Supplementaryfile1.docx]

**SUPPLEMENTARY MATERIAL**

**Table S1** Extreme late-onset drug-level TTO observation among ICI-exposed strict MMM overlap reports (maximum observed TTO).

| **ICI class** | **START_ICI** | **EVENT_DATE2 (source)** | **Drug-level TTO, days** | **Reported death outcome** |
| --- | --- | --- | --- | --- |
| PD-L1 | 2020-10-15 | 2025-05-16 (MFR_DT) | 1,674 | No |

**Abbreviations:** MMM, myocarditis–myositis–myasthenia gravis; TTO, time-to-onset; ICI, immune checkpoint inhibitor. **Notes:** FAERS report identifiers were removed for anonymization.

**Table S2** PS-only sensitivity analyses.

| **Outcome** | **Analysis** | **Result** |
| --- | --- | --- |
| Disproportionality | MMM overlap vs non-overlap | ROR 236.86 (95% CI 164.33–341.40) |
| Drug-level TTO | All valid TTO (TTO ≥0 days) Sensitivity: TTO ≤365 days | n=51; median 24 (IQR 18–37) n=48; median 23 (IQR 18–34) |
| Weibull shape (β) | All valid TTO (TTO >0) Sensitivity: TTO ≤365 days (TTO >0) | β=0.64 (bootstrap 95% CI 0.55–1.34) β=1.37 (bootstrap 95% CI 1.18–1.97) |
| Bayesian disproportionality | IC, MMM overlap vs non-overlap | IC 5.59 (IC025 5.31) |

**Abbreviations:** MMM, myocarditis–myositis–myasthenia gravis; ICI, immune checkpoint inhibitor; PS, primary suspect; ROR, reporting odds ratio; TTO, time-to-onset; CI, confidence interval; IQR, interquartile range; IC, information component; IC025, lower 95% credibility limit of IC.
**Cell definitions (2×2):** a=113, b=186,701, c=38, d=15,000,459 (a: ICI-exposed MMM; b: ICI-exposed non-overlap; c: Non-ICI MMM; d: Non-ICI non-overlap).

**Table S3** Reported death outcome (DE) by exposure group in strict MMM overlap reports (PS-only sensitivity).

| **Exposure group** | **Death / Total** | **Reported death outcome %** |  |
| --- | --- | --- | --- |
| PD-1 | 33 / 86 | 38.4 |  |
| PD-L1 | 12 / 21 | 57.1 |  |
| CTLA-4 | 1 / 6 | 16.7 |  |
| Non-ICI | 8 / 38 | 21.1 |  |

**Abbreviations:** MMM, myocarditis–myositis–myasthenia gravis; ICI, immune checkpoint inhibitor; DE, death.
**Notes:** Percentages are descriptive and should be interpreted cautiously given sparse counts in some strata, especially CTLA-4. Differences in CTLA-4 counts between PS-only and PS/SS analyses reflect domain-specific ICI class assignment. No combination-ICI category was observed under the PS-only domain-specific class assignment; therefore, only PD-1, PD-L1, CTLA-4, and Non-ICI groups are shown in Table S3.

**Table S4** Class-specific ROR and IC for MMM overlap (ICI class vs Non-ICI)

| **ICI class** | **a** | **b** | **c** | **d** | **ROR** | **95% CI** | **IC** | **IC025** |
| --- | --- | --- | --- | --- | --- | --- | --- | --- |
| PD-1 | 80 | 148,713 | 20 | 14,945,845 | 394.65 | 243.01–640.93 | 5.76 | 5.43 |
| PD-L1 | 16 | 52,094 | 20 | 14,945,845 | 230.92 | 120.76–441.58 | 4.72 | 3.93 |
| Combo | 35 | 33,040 | 20 | 14,945,845 | 783.34 | 454.75–1,349.34 | 5.84 | 5.32 |

**Abbreviations:** MMM, myocarditis–myositis–myasthenia gravis; ICI, immune checkpoint inhibitor; ROR, reporting odds ratio; CI, confidence interval; IC, information component; IC025, lower 95% credibility limit of IC.
**Cell definitions (2×2):** *a*, ICI-exposed MMM overlap; *b*, ICI-exposed non-overlap; *c*, non-ICI MMM overlap; *d*, non-ICI non-overlap.
**Notes:** CTLA-4 monotherapy cases did not meet criteria for strict MMM overlap under the PS/SS definition; therefore, class-specific ROR could not be estimated. As a complementary Bayesian disproportionality metric, the Information Component (IC) and its lower 95% credibility limit (IC025) were calculated; a positive Bayesian signal was defined as IC025 > 0.

**Table S5** Demographic characteristics, reporter type, reporting region, and serious outcomes among ICI-exposed MMM overlap reports (n=131)

| **Variable** | **Category** | **n (%)** |
| --- | --- | --- |
| Age category | <50 50–64 ≥65 Unknown | 2 (1.5) 13 (9.9) 89 (67.9) 27 (20.6) |
| Sex | Male Female Unknown | 81 (61.8) 37 (28.2) 13 (9.9) |
| Reporter type | Healthcare professional Consumer/non-healthcare Unknown | 117 (89.3) 11 (8.4) 3 (2.3) |
| Reporting region | US Non-US | 53 (40.5) 78 (59.5) |
| Serious outcomes | Hospitalization Life-threatening Death | 97 (74.0) 53 (40.5) 49 (37.4) |

**Abbreviations:** MMM, myocarditis–myositis–myasthenia gravis; ICI, immune checkpoint inhibitor.
**Notes:** Values are shown as n (%). Percentages are calculated over the total sample of ICI-exposed strict MMM overlap reports (n = 131). Reporter type was derived from OCCP_COD and grouped as healthcare professional, consumer/non-healthcare, or unknown. Reporting region was categorized as US versus Non-US based on REPORTER_COUNTRY. Serious outcomes were derived from FAERS OUTC codes.

**Table S6** Selected published clinical literature on ICI-associated MMM overlap and related overlap-spectrum phenotypes

| **Study (Ref.)** | **Design** | **Population / tumor type / ICI regimen** | **Key clinical details (onset, presentation, management, outcome)** | **Relevance to the present analysis** |
| --- | --- | --- | --- | --- |
| **Lipe et al. (8)** | Systematic review | 50 published cases of ICI-associated MMM overlap; most commonly melanoma, lung cancer, and renal cell carcinoma; mixed ICI regimens | Median time from ICI initiation to MMM presentation was 21 days; strict triad phenotype; in-hospital mortality was 38.0%; detailed case-level descriptions, but evidence was derived predominantly from case reports and small case series | Provides the most directly comparable clinical benchmark for the early-onset pattern and reported death outcome burden observed in our FAERS analysis |
| **Sánchez-Camacho et al. (9)** | Single-institution case series with literature review | 4 patients with ICI-associated MMM overlap; mixed solid tumors and ICI regimens | Presentation involved combined cardiac and neuromuscular toxicity; emphasized early multidisciplinary evaluation; corticosteroids were first-line, with escalation to IVIG, plasma exchange, and selected second-line immunomodulatory therapies in severe cases | Supports the clinical management implications of MMM overlap and highlights the absence of standardized treatment algorithms |
| **Itzhaki Ben Zadok et al. (10)** | Retrospective cohort study | Patients with ICI-related myocarditis, with or without concomitant immune-related myopathy; mixed tumor types and ICI regimens | Concomitant immune-related myopathy was associated with distinct biomarker profiles and a greater likelihood of clinically significant arrhythmias; overlap phenotypes appeared to have greater cardiologic instability | Supports the concept that overlap-spectrum phenotypes may carry a particularly unstable cardiac profile and may help explain the clinical seriousness of MMM overlap |
| **Plomp et al. (11)** | Multicentric retrospective cohort study | Patients with ICI-treated myositis and myasthenic features; multicenter real-world cohort; mixed tumor types and regimens | Demonstrated that myositis and myasthenic manifestations frequently coexist in clinical practice; overlap-spectrum presentations were not limited to isolated anecdotal reports; management was heterogeneous across centers | Helps position MMM within a broader overlap-spectrum literature and supports the view that these toxicities extend beyond isolated case reports |

**Abbreviations:** FAERS, FDA Adverse Event Reporting System; ICI, immune checkpoint inhibitor; IVIG, intravenous immunoglobulin; MMM, myocarditis–myositis–myasthenia gravis.


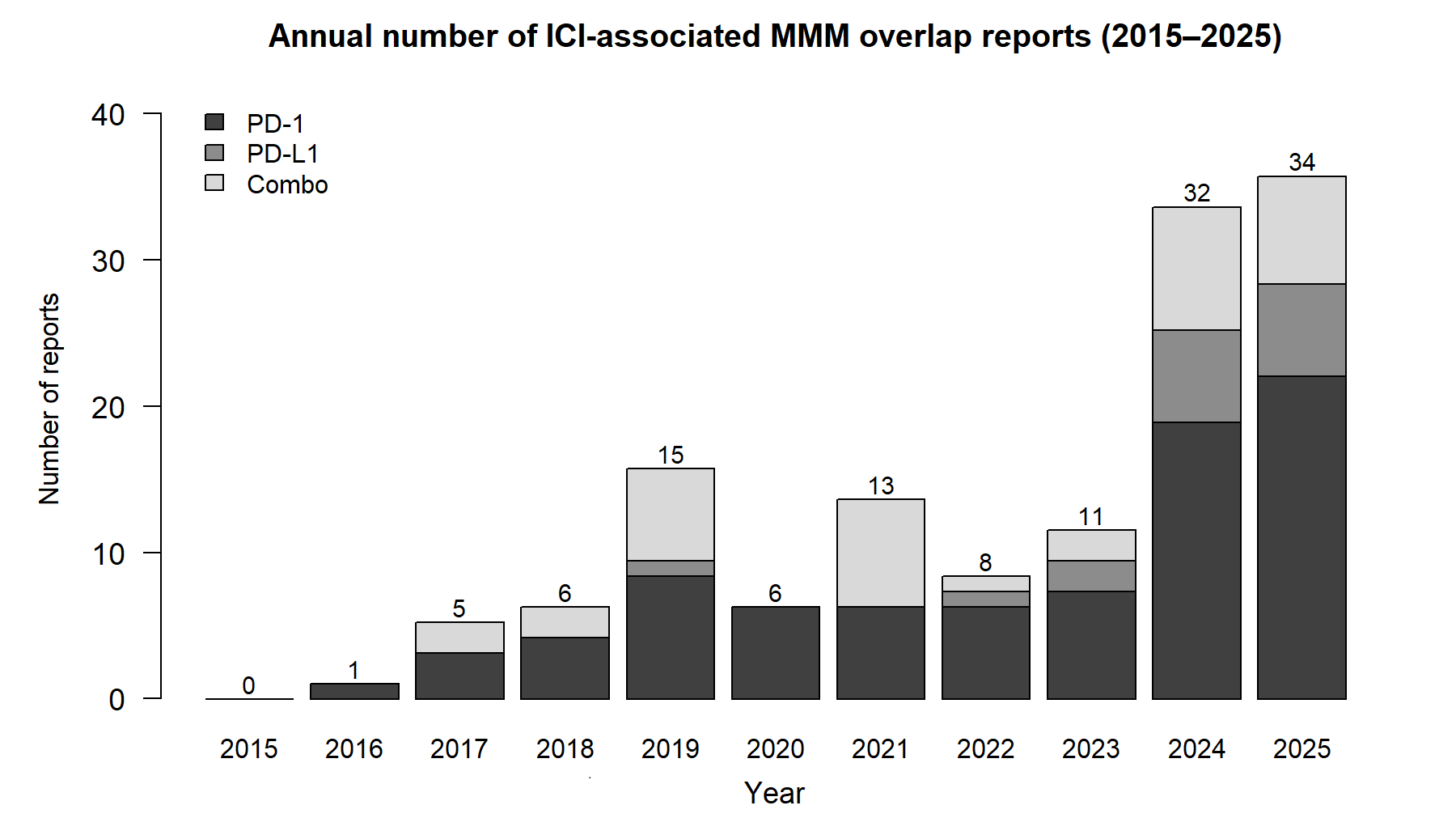


**Figure S1** Annual number of MMM overlap reports associated with ICIs (2015–2025)

**Abbreviations:** MMM, myocarditis–myositis–myasthenia gravis; ICI, immune checkpoint inhibitor. **Notes:** No CTLA-4 monotherapy MMM overlap reports were observed under the PS/SS definition in this dataset and are therefore not displayed.
